# Supplementary material for: Deletion of neural estrogen receptor alpha induces sex differential effects on reproductive behavior in mice
Source: Commun Biol. 2022 Apr 20;5:383. doi: 10.1038/s42003-022-03324-w (PMC9021208; doi:10.1038/s42003-022-03324-w)
Supplement: Supplementary file 2 — Supplementary Information [file 42003_2022_3324_MOESM2_ESM.pdf]

Title: Deletion of neural estrogen receptor alpha induces sex differential effects on reproductive behavior in mice

Authors:

Anne-Charlotte Trouillet, Suzanne Ducroq, Lydie Naulé, Daphné Capela, Caroline Parmentier, Sally Radovick, Hélène Hardin-Pouzet, Sakina Mhaouty-Kodja

SUPPLEMENTARY INFORMATION

Content:

Supplementary Figure 1, relative to Figure 1

Supplementary Figure 2

Supplementary Figure 3

Supplementary Figure 4

Supplementary Table 1

Supplementary Table 2

Supplementary Figure 1, relative to Figure 1.Uncropped gels.

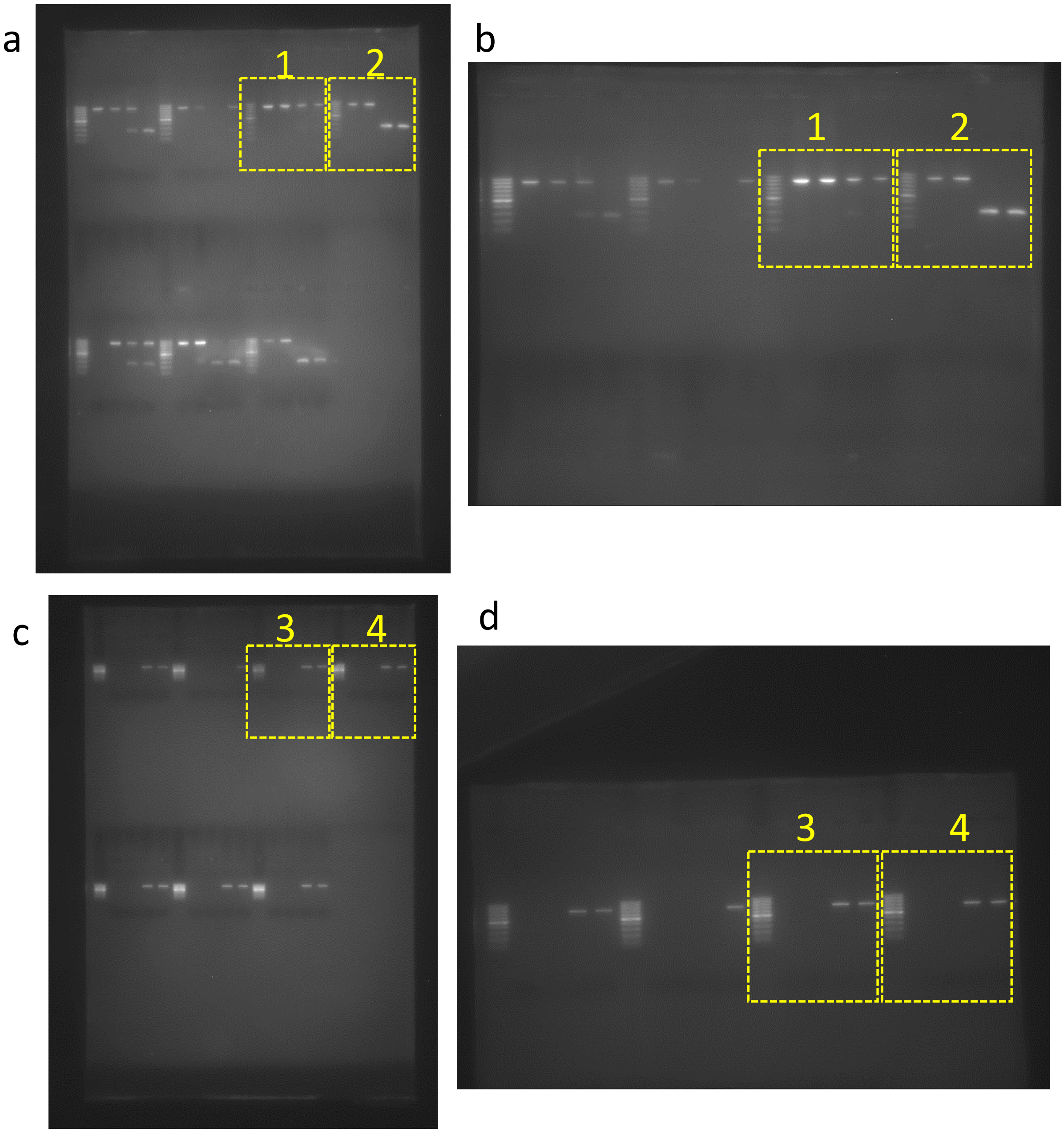

The original gel (a and c) and the original gel magnification (b and d) used to construct the figure 1a. The square labeled 1 correspond the upper left panel, the square labeled 2 to the upper right panel, the square labeled 3 to the lower left panel, and the square labeled 4 to the lower right panel.

Supplementary Figure 2. Assessment of locomotor activity and anxiety-related behavior in gonadectomized and testosterone-supplemented ER^{NesCre} males.

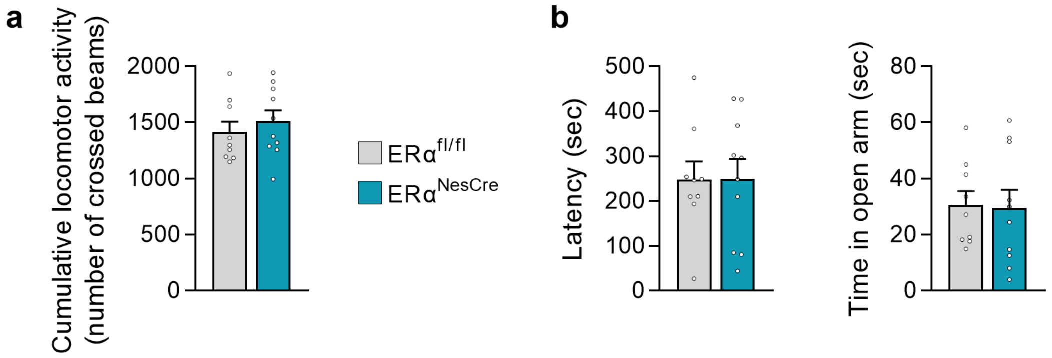

a. Locomotor activity during the 2 h-test for control (ERα$^{fl/fl}$) and mutant (ERα^{NesCre}) males (n = 9-10 per genotype). **b.** *Left*. Latency to enter the open arms of the O-maze. *Right*. Time spent in the open arms. All data are shown as means ± S.E.M with individual values.

Supplementary Figure 3. Delayed puberty onset in ER^{NesCre} females.

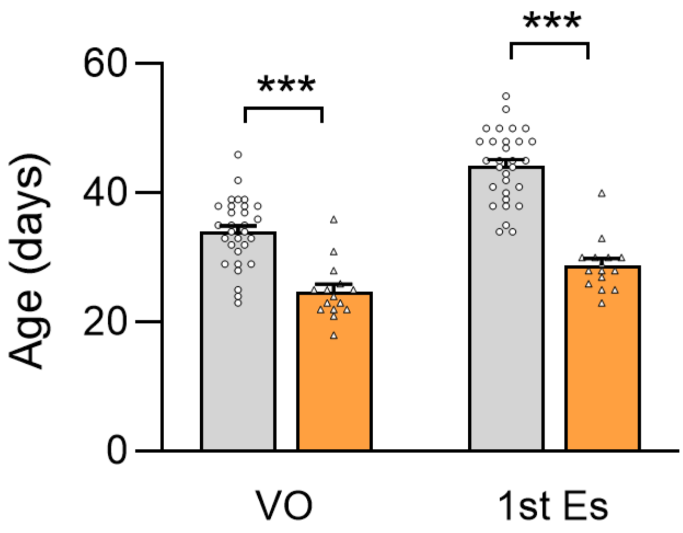

Age at vaginal opening (VO) and first estrus (1st Es) of ER$^{fl/fl}$ and ER^{NesCre} females (n = 15-30 per genotype). ***$p < 0.001$ versus control (ERα$^{fl/fl}$) animals. Data are shown as means ± S.E.M with individual values.

Supplementary Figure 4. Behavior of male stimuli during the lordosis behavior assessment of ER^{NesCre} females.

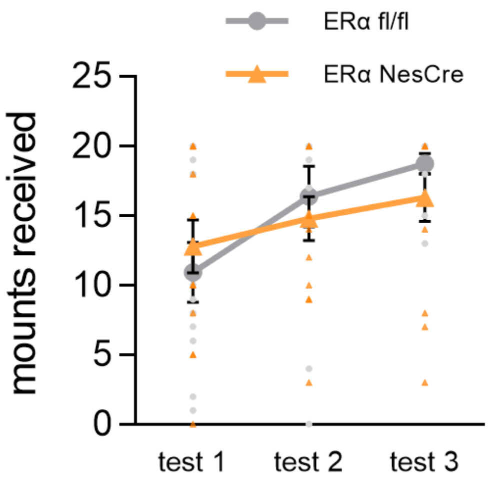

Number of mounts received during the three lordosis behavior tests of ER$^{fl/fl}$ and ER^{NesCre} females (n = 11-13 per genotype). Male stimuli gave a similar amount of mounts to control and mutant females. Data are shown as means ± S.E.M with individual values.

Supplementary Table 1. Analysis of physiological parameters in (ERα$^{fl/fl}$) and mutant (ERα^{NesCre}) females and males.

Genotype		ERα$^{fl/fl}$	ERα^{NesCre}
Females	Body weight (bw) (g)	23.99 ± 0.62	22.58 ± 0.49
	Ovary weight (%bw)	0.04 ± 0.001	0.03 ± 0.002**
	Uterus weight (%bw)	0.27 ± 0.01	0.73 ± 0.07***
	Estradiol levels (pg/ml)	46.61 ± 7.58	75.29 ± 9.28*
	Number of corpora lutea	2.45 ± 0.53	0
Males	Body weight (bw) (g)	30.72 ± 0.74	29. 04 ± 0.81*
	Seminal vesicle weight (%bw)	1.04 ± 0.05	1.25 ± 0.05**
	Testis weight (%bw)	0.65 ± 0.01	0.65 ± 0.02
	Testosterone levels (ng/ml)	5.51 ± 1.71	11.01 ± 1.52*
	Estradiol levels (pg/ml)	13.86 ± 1.95	8.39 ± 1.67*
	Sperm Count (10^6/mL)	74.95 ± 9.27	77.31 ± 9.28

Data are means ± S.E.M. for 11-12 females per genotype for body and organs weights, 7-9 females per genotype for estradiol levels, 3 females per genotype for the number of corpora lutea, 22 to 24 males per genotype for body, seminal vesicles and testis weights, 8-11 males per genotype for testosterone levels, 10-13 males per genotype for estradiol levels and 8 to 10 males per genotype for sperm count. *$p < 0.05$, **$p < 0.01$; ***$p < 0.001$ versus control (ERα$^{fl/fl}$) animals.

Supplementary Table 2. Analysis of reproductive parameters of control (AR::ERα$^{fl/fl}$) and mutant (AR::ERα^{NesCre}) males.

**Genotype**	**AR::ERα$^{fl/fl}$**	**AR::ERα^{NesCre}**
Body weight (bw) (g)	31.67 ± 1.31	26.97 ± 0.69*
Seminal vesicle weight (%bw)	0.95 ± 0.03	1.49 ± 0.18***
Testis weight (%bw)	0.52 ± 0.04	0.42 ± 0.04
Testosterone levels (ng/ml)	2.36 ± 0.57	4.71 ± 0.91*

Data are means ± S.E.M. for 10-17 males per genotype for body and organ weight and 8-10 for testosterone levels. *$p < 0.05$, ***$p < 0.001$ versus control (ERα$^{fl/fl}$) animals.
