# Supplementary material for: Deletion of neural estrogen receptor alpha induces sex differential effects on reproductive behavior in mice
Source: Commun Biol. 2022 Apr 20;5:383. doi: 10.1038/s42003-022-03324-w (PMC9021208; doi:10.1038/s42003-022-03324-w)
Supplement: Supplementary file 5 — Reporting Summary [file 42003_2022_3324_MOESM5_ESM.pdf]

nature portfolio

Corresponding author(s): Mhaouty-Kodja Sakina

Last updated by author(s): March 24, 2022

Reporting Summary

Nature Portfolio wishes to improve the reproducibility of the work that we publish. This form provides structure for consistency and transparency in reporting. For further information on Nature Portfolio policies, see our Editorial Policies and the Editorial Policy Checklist.

Statistics

For all statistical analyses, confirm that the following items are present in the figure legend, table legend, main text, or Methods section.

n/a	Confirmed	
☐	☒	The exact sample size (n) for each experimental group/condition, given as a discrete number and unit of measurement
☐	☒	A statement on whether measurements were taken from distinct samples or whether the same sample was measured repeatedly
☐	☒	The statistical test(s) used AND whether they are one- or two-sided<br>*Only common tests should be described solely by name; describe more complex techniques in the Methods section.*
☐	☒	A description of all covariates tested
☐	☒	A description of any assumptions or corrections, such as tests of normality and adjustment for multiple comparisons
☒	☐	A full description of the statistical parameters including central tendency (e.g. means) or other basic estimates (e.g. regression coefficient) AND variation (e.g. standard deviation) or associated estimates of uncertainty (e.g. confidence intervals)
☒	☐	For null hypothesis testing, the test statistic (e.g. F, t, r) with confidence intervals, effect sizes, degrees of freedom and P value noted<br>*Give P values as exact values whenever suitable.*
☒	☐	For Bayesian analysis, information on the choice of priors and Markov chain Monte Carlo settings
☒	☐	For hierarchical and complex designs, identification of the appropriate level for tests and full reporting of outcomes
☒	☐	Estimates of effect sizes (e.g. Cohen's d, Pearson's r), indicating how they were calculated

Our web collection on statistics for biologists contains articles on many of the points above.

Software and code

Policy information about availability of computer code

Data collection	Not applicable
Data analysis	Not applicable

For manuscripts utilizing custom algorithms or software that are central to the research but not yet described in published literature, software must be made available to editors and reviewers. We strongly encourage code deposition in a community repository (e.g. GitHub). See the Nature Portfolio guidelines for submitting code & software for further information.

Data

Policy information about availability of data

All manuscripts must include a data availability statement. This statement should provide the following information, where applicable:

- Accession codes, unique identifiers, or web links for publicly available datasets
- A description of any restrictions on data availability
- For clinical datasets or third party data, please ensure that the statement adheres to our policy

Not applicable

Field-specific reporting

Please select the one below that is the best fit for your research. If you are not sure, read the appropriate sections before making your selection.

☒ Life sciences ☐ Behavioural & social sciences ☐ Ecological, evolutionary & environmental sciences

For a reference copy of the document with all sections, see nature.com/documents/nr-reporting-summary-flat.pdf

Life sciences study design

All studies must disclose on these points even when the disclosure is negative.

Sample size	The sample size for studies was determined on the basis of our previous experience in the field, to avoid inconsistent interpretation due to an inappropriate number of animals. Behavioral studies are usually conducted on a mean number of at least 7 animals per group. The immunohistochemical studies were conducted on at least 5 animals per group.
Data exclusions	No data exclusions
Replication	The female phenotype for ERalpha deletion was confirmed by several combined approaches (Immunohistochemistry of key hypothalamic neuronal populations, behavioral analyses, physiological parameters (presence of corpora lutea and hormonbal dosage), estrous cyclicity. The male phenotype for ERalpha deletion was also chkecked using several approaches (behavior, immunohistochemistry, urogenital tract measures, hormonal levels...). In addition, both intact and gonadectomized hormpnally-primed males were analyzed.
Randomization	Groups used in the same study were composed of control and mutant littermates derived from different litters, with a mean age difference of no more than 1 to 1.5 months.
Blinding	The analyses were performed by blind observation as described in the "Methods" section.

Reporting for specific materials, systems and methods

We require information from authors about some types of materials, experimental systems and methods used in many studies. Here, indicate whether each material, system or method listed is relevant to your study. If you are not sure if a list item applies to your research, read the appropriate section before selecting a response.

Materials & experimental systems

n/a	Involved in the study	
☐	☒	Antibodies
☒	☐	Eukaryotic cell lines
☒	☐	Palaeontology and archaeology
☐	☒	Animals and other organisms
☒	☐	Human research participants
☒	☐	Clinical data
☒	☐	Dual use research of concern

Methods

n/a	Involved in the study	
☒	☐	ChIP-seq
☒	☐	Flow cytometry
☒	☐	MRI-based neuroimaging

Antibodies

Antibodies used	The primary antibodies used are:<br>Rabbit anti-androgen receptor (sc-816, Santa Cruz Biotechnology)<br>Mouse anti-tyrosine hydroxylase (MAB318, Millipore)<br>Mouse anti-calbindin (C9848, Sigma-Aldrich)<br>Sheep anti-kisspeptin (AC053, generous gift of I. Franceschini, Franceschini et al., 2013)<br>Rabbit anti-progesterone receptor (A0098, Dako)
Validation	The anti-androgen receptor is a widely used specific antibody, which was validated by our previous studies on control and neural deleted AR knockout mice, showing no specific signal in the brain of mutant males.<br>The anti-kisspeptin is a very specific antibody devloped by A. Caraty (the referecnce is cited in the "Methods" section). This antibody is known and used by the researchers working on the regulation of the gonadotrope axis by kisspeptin neurons.<br>The other antibodies are widely used specfic antibodies highly described in the literature.

Animals and other organisms

Policy information about studies involving animals; ARRIVE guidelines recommended for reporting animal research

Laboratory animals	The study used both male and female mice of the ERalphaNesCre mouse line generated on on a mixed C57BL/6 x CD1 background, as described in the text. The age of animals was comprised between 3 and 6 months.
Wild animals	Not applicable
Field-collected samples	Not applicable
Ethics oversight	The experimental protocols were approved by the “Charles Darwin” Ethical committee (project number 01490-01).

Note that full information on the approval of the study protocol must also be provided in the manuscript.
